# The impact of nucleic acid testing to detect human immunodeficiency virus, hepatitis C virus, and hepatitis B virus yields from a single blood center in China with 10-years review

**DOI:** 10.1186/s12879-022-07279-5

**Published:** 2022-03-23

**Authors:** Danxiao Wu, Fangjun Feng, Xiaojuan Wang, Dairong Wang, Yiqin Hu, Yang Yu, Jihong Huang, Min Wang, Jie Dong, Yaling Wu, Hong Zhu, Faming Zhu

**Affiliations:** 1 Blood Center of Zhejiang Province, Jianye Road 789, Hangzhou, Zhejiang 310052 People’s Republic of China; 2 Key Laboratory of Blood Safety Research of Zhejiang Province, Jianye Road 789, Hangzhou, Zhejiang 310052 People’s Republic of China

**Keywords:** Nucleic acid amplification test, Blood screening, Detection capacity, NAT yields

## Abstract

**Background:**

Since 2010, the Blood Center of Zhejiang province, China, has conducted a pilot nucleic acid amplification testing (NAT) screening of blood donors for Hepatitis B virus (HBV), Hepatitis C virus (HCV), and Human immunodeficiency virus (HIV). This study aims to assess the results of NAT testing over 10 years to establish the effects and factors influencing NAT yields of HBV, HCV, and HIV.

**Methods:**

Blood donations from seven different blood services were screened for HBV DNA, HCV RNA, and HIV RNA using 6 mini pools (6MP) or individual donation (ID)-NAT method between August 1, 2010, and December 31, 2019, at the NAT centralized screening center. We compared 3 transcription-mediated amplification (TMA) assays and 2 polymerase chain reaction (PCR) assays. Further, HBV, HCV, and HIV NAT yields were calculated and donor characteristics and prevalence of HBV NAT yields analyzed. Donors with HCV and HIV NAT yield were also followed up.

**Results:**

1916.31 per million donations were NAT screening positive overall. The NAT yields for HBV, HCV, HIV and non-discriminating reactive were 1062.90 per million, 0.97 per million, 1.45 per million, and 850.99 per million, respectively, which varied in the seven blood services and different years. HBV NAT yields were higher than those of HCV and HIV and varied across demographic groups. Risk factors included being male, old age, low education level, and first-time donors. We found no differences in NAT yields of HBV, HCV, and HIV between the 3 TMA and 2 PCR assays; nonetheless, statistically, significant differences were noted between the five assays.

**Conclusion:**

In summary, NAT screening in blood donations reduces the risk of transfusion-transmitted infections and shortens the window period for serological marker screening. Therefore, a sensitive NAT screening method, ID-NAT workflow, and recruitment of regular low-risk donors are critical for blood safety.

**Supplementary Information:**

The online version contains supplementary material available at 10.1186/s12879-022-07279-5.

## Backgrounds

Blood transfusion saves millions of lives annually across the globe. Nonetheless, transfusion transmissible infections (TTIs) remain a major problem. The main TTIs include hepatitis B virus (HBV), hepatitis C virus (HCV), human immunodeficiency virus (HIV), and *Treponema pallidum* (TP) [1–3]. Notably, HIV, HBV, and HCV are causative agents of AIDS, hepatitis B, and C infection, respectively. Regardless of the low viral load, the risk of transmitting these viruses through transfusion of infected blood is markedly higher than through other routes [4]. The prevalence of these viral infections among blood donors varies by geography and nationality; it directly hinges on their prevalence in the general population. Based on the global estimates by the WHO (World Health Organization) till 2015, HBV and HCV chronically infected 257 million people and 71 million people, respectively. By the end of 2019, 38 million individuals were newly infected by HIV. Nevertheless, the prevalence of HBV, HCV, and HIV infections among blood donors in different countries and world regions varies from 0.003 to 5.54%, 0.002 to 2.23%, and 0.00 to 1.66%, respectively. For over 10 years, chronic hepatitis B is the leading among 27 infectious diseases reported by the Chinese government. Approximately 50% of the Chinese population has a history of HBV infection, out of which, 7.18% are chronic carriers of hepatitis B surface antigen (HBsAg) [5, 6]. Therefore, HBV is a major threat to blood safety in China.

Of note, advances in molecular screening for TTIs have significantly reduced the risk of infection transmission via blood transfusion. Nucleic acid amplification testing (NAT) is used to diagnose viral infections in transfusion medicine and is mandatory for blood services in China since 2016. The benefits of NAT include the capacity to directly detect viral genomes (DNA or RNA) with high specificity. Its sensitivity is several orders of magnitude greater than that of antigen and/or antibody immunological assays. Besides, NAT has markedly reduced the assay window for immunological assays [7]. In the present study, we assess the results of NAT over 10 years and analyze their effects on blood safety at the Blood Center of Zhejiang Province, China, where the infection rate of HBV is higher than that in the general population.

## Methods

### Blood sample collection

Nucleic acid amplification testing (NAT) centralized screening policy was implemented in Zhejiang Province, China, and the Blood Center of Zhejiang province, one of the centralized screening sites. Study samples were respectively collected from voluntary unpaid donors at the Blood Center of Zhejiang Province, and Xiaoshan, Jiande, Yiwu, Shaoxing, Jiaxing, and Huzhou blood stations, between August 1, 2010, and December 31, 2019. The Blood Center of Zhejiang province is located in the Hangzhou region; Xiaoshan and Jiande are counties in the Hangzhou region. Thus, blood donors from the Blood Center of Zhejiang Province were divided into three regions, including Hangzhou, Xiaoshan, and Jiande. During the implementation of Zhejiang Province’s NAT centralized screening policy, the start time for NAT detection varied depending on the blood service center. NAT was used from August 1, 2010, at the Blood Center of Zhejiang province; from May 29, 2013, at Xiaoshan and Jiande; from September 5, 2013, at Yiwu; and from March 1, 2016, at Shaoxing, Jiaxing, and Huzhou. All samples were collected, stored, and handled following the manufacturer’s instructions after obtaining informed consent from blood donors.

### Pre- and post-donation screening of blood donors

Based on the guidelines for blood donation in China, the donors filled in a risk factor questionnaire excluding those at risk of exposure to transfusion transmissible infections. Safe donors were physically examined by a doctor before acceptance for donation. Thereafter, the donors underwent pre-donation screening, including determination of ABO blood group, hemoglobin concentration, ALT level, and HBsAg status. Donors with low hemoglobin concentration (male: < 120 g/L; females:  < 110 g/L before July 1, 2012, or  < 115 g/L from July 1, 2012 due to a policy change), abnormal ALT level (> 40 IU/L before July 1, 2012, and > 50 IU/L since July 1, 2012, due to a policy change) or positive HBsAg results were temporarily deferred.

After donation, blood samples were tested for ALT level and ABO type then screened for HBsAg, anti-HCV, anti-HIV, and anti-TP using 2 ELISA kits from different manufacturers (Additional file 1: Table S1). Reactive samples on either kit for any viral marker were defined as positive for that marker (ELISA^+^). Assays were conducted as per the manufacturer’s instructions.

### Nucleic acid amplification testing (NAT) assays

The HBV, HCV, and HIV NAT assays were run in parallel for the relevant donor samples using 6 mini pools NAT (6MP-NAT, Roche Diagnostics, Manheim, Germany) or individual NAT (ID-NAT, Novartis Diagnostics, Emeryville, CA, USA) modes, based on the manufacturer’s instructions (Table 1). The workflow for ID-NAT using a transcription-mediated amplification (TMA) was performed on initially positive blood donations retested in parallel using a similar ID-NAT screening and discriminatory assays, leading to two types of results, i.e., positive screening tests but non-discriminating, or results that discriminate between HBV, HCV or HIV. Nonetheless, all were defined as positive. Donated blood was analyzed using individual NAT to whether they were reactive in the 6MP-NAT mode, yielding positive or negative results on individual NAT confirmatory tests for utilizing the TaqMan PCR platform. NAT^+^ELISA^−^ donors should be deferred according to the guideline in China.

**Table 1:** NAT reagents used for screening donors in different methods and systems

System			Procleix® Tigris® system		Procleix® Panther® system		cobas s 201 system	
Methods			TMA, individual NAT for screening and discriminatory assays				TaqMan PCR, 6 mini pool NAT for screening assay and individual NAT for confirmatory assay	
Time range for using			August 1, 2010- July 31, 2015	August 1, 2015- September 21, 2016	September 22, 2016- December 31, 2019		April 9, 2013- November 30, 2013	December 1, 2013- December 31, 2019
Kit name (Company)			Procleix® Ultrio® Assay (Novartis Diagnostics, Emeryville, CA, USA)	Procleix® Ultrio Plus® Assay (Novartis Diagnostics, Emeryville, CA, USA)	Procleix® Ultrio Elite® Assay (Novartis Diagnostics, Emeryville, CA, USA)		Cobas® TaqScreen MPX Test (Roche Diagnostics, Manheim, Germany)	Cobas® TaqScreen MPX Test, version 2.0 (Roche Diagnostics, Manheim, Germany)
Sensitivity (IU/mL, 95%LOD)	HBV	ID-NAT	10.4 (9.2–12.2)	3.4 (3.0–4.1)	4.3 (3.8–5.0)	HBV	3.8 (3.3–4.4)	2.3 (2.0–2.8)
		dHBV	8.5 (7.6–9.8)	4.1 (3.5–4.9)	4.5 (4.0–5.3)			
	HCV	ID-NAT	3.0 (2.7–3.4)	5.4 (4.5–6.7)	3.0 (2.5–3.9)	HCV	11 (7.0–21.7)	6.8 (5.8–8.3)
		dHCV	3.2 (2.8–3.6)	4.4 (3.7–5.6)	2.4 (2.0–3.3)			
	HIV-1	ID-NAT	47.9 (43.3–54.5)	21.2 (18.2–25.7)	18.0 (15.0–23.5)	HIV-1 M	49 (42.4–58.1)	50.3 (43.3–59.9)
		dHIV	53.6 (47.9–61.2)	18.9 (16.3–22.9)	17.3 (14.4–22.6)	HIV-1 O*	89 (56–217)	18.3 (13.0–31.7)
	HIV-2	ID-NAT	/	/	10.4 (8.9–12.6)	HIV-2*	59.3 (51.9–69.7)	57.4 (49.7–68.1)
		dHIV	/	/	9.6 (8.1–11.8)	HIV-2	/	7.9 (5.6–13.8)

The sensitivity of the cobas201 system is from the individual NAT mode

*HBV* Hepatitis B virus, *HCV* Hepatitis C virus, *HCV-Ab* antibody to hepatitis C virus, *HIV* human immunodeficiency virus, *dHBV* discriminatory test for HBV DNA, *dHCV* discriminatory test for HCV RNA, *dHIV* discriminatory test for HIV RNA, *ID* individual donation, *MP* mini pool, *IU* international unit, *LOD* limit of detection, *NAT* nucleic acid amplification testing, *PCR* polymerase chain reaction, *TMA* transcription-mediated amplification

^*^The unit is Copies/mL

### Comparison of two NAT systems for detection of low viral load level OBI samples

Partial NAT^+^/ELISA^−^ samples were collected between May 1, 2017 and May 1, 2018. Out of these, 103 samples had previously tested positive in non-discriminating reaction, whereas 39 were HBV DNA positive. Anti-HBc was detected via electroluminescence on a Cobas e601 analyzer (Roche Diagnostics Company, Shanghai, China). Viral load was established on a Roche Cobas AmpliPrep with RT-PCR performed on a Cobas TaqMan analyzer (Roche Diagnostics Company, Shanghai, China). Samples were tested thrice on ID-NAT mode using these systems to compare the Ultrio Elite and MPX 2.0 NAT systems; the results were considered positive if at least one test was positive.

### Supplementary assays and follow-up study

Anti-HIV reactive samples were confirmed by Western blot assay at the Centre of Disease Control, Hangzhou, Zhejiang province as per China’s state regulations.

Blood donors positive for HCV or HIV after NAT yet negative by ELISA (NAT^+^/ELISA^−^) were followed up and subjected to tests by serology and NAT.

### Statistical analysis

Statistical analyses were performed on the SPSS 22.0 software. Differences in the rates across various blood services were analyzed using the chi-square test and Fisher’s exact tests, as appropriate. *P* < 0.05 was considered statistically significant.

## Results

### Overall NAT yield rates are various in the difference blood services

A total of 2,071,695 blood donations were NAT screened between August 1, 2010, and December 31, 2019 at Hangzhou, Xiaoshan, Jiande, Yiwu, Shaoxing, Jiaxing, and Huzhou blood service centers. Among these, 1,160,355 (56.01%) were analyzed on ID-NAT mode using the TMA method; the remaining 911,340 (43.99%) were analyzed using the TaqMan PCR method on 6MP-NAT mode. All the NAT yields (NAT^+^/ELISA^−^) cases are shown in Table 2. The overall NAT yield rate was 1916.31 per million. NAT yields rates for HBV, HCV, HIV and non-discriminating reactive were 1062.90 per million, 0.97 per million, 1.45 per million, and 850.99 per million, respectively. Notably, NAT yields rates differed across the 7 blood service centers (χ^2^ = 514.27, *p* < 0.01), with the highest yield at Jiande (4579.84 per million) and the lowest yield at the Jiaxing (1450.31 per million).

**Table 2:** Numbers and proportions of donations in NAT detection and the results of NAT yields in the seven blood services

		Blood services							Method		Overall
		Hangzhou	Xiaoshan	Jiande	Yiwu	Shaoxing	Jiaxing	Huzhou	TMA	PCR	
Years	2010	46,853	/	/	/	/	/	/	46,853	/	46,853
	2011	131,594	/	/	/	/	/	/	131,594	/	131,594
	2012	125,656	/	/	/	/	/	/	125,656	/	125,656
	2013	129,668	479	291	2317	/	/	/	105,668	27,087	132,755
	2014	133,738	13,680	11,844	11,378	/	/	/	119,301	51,339	170,640
	2015	135,470	13,546	12,724	11,410	/	/	/	106,505	66,645	173,150
	2016	139,646	13,846	12,865	13,111	41,526	41,699	27,080	106,494	183,279	289,773
	2017	143,769	14,110	12,419	13,797	48,194	50,571	32,194	158,791	156,263	315,054
	2018	149,465	15,749	13,085	15,412	50,515	54,843	33,291	136,568	195,792	332,360
	2019	161,135	16,400	13,849	15,896	52,741	59,739	34,100	122,925	230,935	353,860
Overall	Numbers	1,296,994	87,810	77,077	83,321	192,976	206,852	126,665	1,160,355	911,340	2,071,695
	Proportions	62.61%	4.24%	3.72%	4.02%	9.31%	9.98%	6.11%	56.01%	43.99%	100.00%
Non-discriminating reactive*	Numbers	1056	101	154	184	124	88	56	1763	/	1763
	NAT yields (per million)	814.19	1150.21	1998.00	2208.33	642.57	425.42	442.11	1519.36	/	850.99
HBV	Numbers	1146	109	199	132	264	212	140	1279	923	2202
	NAT yields (per million)	883.58	1241.32	2581.83	1584.23	1368.05	1024.89	1105.28	1102.25	1012.79	1062.90
HCV	Numbers	2	0	0	0	0	0	0	2	0	2
	NAT yields (per million)	1.54	0	0	0	0	0	0	1.72	0	0.97
HIV	Numbers	3	0	0	0	0	0	0	2	1	3
	NAT yields (per million)	2.31	0	0	0	0	0	0	1.72	1.10	1.45
ALL NAT^+^ELISA^−^	Numbers	**2207**	**210**	**353**	**316**	**388**	**300**	**196**	**3046**	**924**	**3970**
	NAT yields (per million)	**1701.63**	**2391.53**	**4579.84**	**3792.56**	**2010.61**	**1450.31**	**1547.39**	**2625.06**	**1013.89**	**1916.31**

Bold values indicate the total NAT positive numbers and NAT yields in different regions

*HBV* Hepatitis B virus, *HCV* Hepatitis C virus, *HIV* Human immunodeficiency virus, *ELISA* enzyme linked immunosorbent assay, *NAT* nucleic acid amplification testing, *PCR* polymerase chain reaction, *TMA* transcription-mediated amplification

^*^The donations were reactive in ID-NAT screening assays but not in discriminatory assays using TMA method

### The difference in NAT yields rates between the TMA and PCR methods

A big gap in NAT yield rate was found in the TMA vs PCR method (2625.06 per million vs 1013.89 per million, χ^2^  = 692.78, *p* < 0.01, Table 3). This gap suggests that 6 mini pools NAT (MP-NAT) exhibit less sensitivity, whereas ID-NAT lacks specificity. HBV NAT yield rates were similar in TMA vs PCR methods, at 1102.25 per million vs 1012.79 per million (*p* > 0.05, Table 3). NAT yields rates of HCV and HIV were higher under the TMA method than that under the PCR method; however, the difference was not statistically significant (*p* values = 0.507 and 1.000, respectively, Fisher’s exact test). Notably, all the NAT yield rates (HBV, HCV, and HIV) in the TMA method were significantly higher than that in the PCR method (χ^2^ = 4.04, *p* < 0.05); this may be attributed to the sensitivity methods and differences in NAT screening modes.

**Table 3:** The results of NAT yields for five different assays using the TMA and PCR methods

Method		TMA				PCR			Overall
Assays		Procleix® Ultrio® Assay	Procleix® Ultrio Plus® Assay	Procleix® Ultrio Elite® Assay	Total	Cobas® TaqScreen MPX Test	Cobas® TaqScreen MPX Test, version 2.0	Total	
Number of tested donations		584,540	131,614	444,201	1,160,355	73,052	838,288	911,340	2,071,695
Non-discriminating reactive	Numbers	797	271	695	1763	/	/	/	1763
	NAT yields (per million)	1363.47	2059.05 **	1564.61 **^##^	1519.36	/	/	/	850.99
HBV	Numbers	444	127	708	1279	66	857	923	2202
	NAT yields (per million)	759.57	964.94 *	1593.87 **^##^	1102.25	**903.47 **^**@@**^	1022.32**^## @@^	1012.79	1062.90
HCV	Numbers	0	2	0	2	0	0	0	0
	NAT yields (per million)	0.00	15.20 *^@@^	0.00	1.72	0.00	0.00	0.00	0.00
HIV	Numbers	1	0	1	2	0	1	1	3
	NAT yields (per million)	1.71	0.00	2.25	1.72	0.00	1.19	1.10	1.45
NAT^+^ELISA^−^ (HBV, HCV and HIV)	Numbers	445	129	709	1283	66	858	924	2207
	NAT yields (per million)	761.28	980.14 *	1596.12 **	1105.70	903.47	1023.51	**1013.89 **^**&**^	1065.31
NAT^+^ELISA^−^ (ALL kinds)	Numbers	1242	400	1404	3046	66	858	924	3970
	NAT yields (per million)	2124.75	3039.19 **	3160.73 **	2625.06	903.47	1023.51	1013.89	1916.31

Bold values indicate significant differences

*HBV* hepatitis B virus, *HCV* hepatitis C virus, *HIV* human immunodeficiency virus, *ELISA* enzyme linked immunosorbent assay, *NAT* nucleic acid amplification testing, *PCR* polymerase chain reaction, *TMA* transcription-mediated amplification

^*^p < 0.05,**p < 0.01, compared to Procleix® Ultrio® Assay; #p < 0.05, ##p < 0.01, compared to Procleix® Ultrio Plus® Assay; @@p < 0.01, compared to Procleix® Ultrio Elite® Assay; &p < 0.05, compared to TMA method

### Comparison of NAT yield rates in the different assays using TMA and PCR methods

Further, we compared the NAT yields rates for the 3 TMA and 2 PCR assays used in this study (Table 3). Analysis of NAT yield rates in the TMA assays revealed that the NAT yield rates of Ultrio Plus and Ultrio Elite assays were higher than those of the Ultrio assay (χ^2^  = 113.19, *p* < 0.01). HBV NAT yield rates were the highest in the Ultrio Elite assay, followed by Ultrio Plus and Ultrio assays (χ^2^  = 162.11, *p* < 0.01). Nonetheless, only 2 HCV NAT yield cases were found by the Ultrio Plus assay (*p* < 0.05, Fisher’s exact test). Moreover, we found two HIV NAT yields individuals using the TMA method. Differences in HIV NAT yield rates were not statistically significant in the 3 TMA assays.

NAT yield rates of HBV, HCV, and HIV did not significantly differ between the MPX and MPX2.0 PCR methods (χ^2^ = 0.96, *p* > 0.05). Notably, a comparison of HBV NAT yield rates across the 5 assays revealed that HBV NAT yields rate is lower in the MPX assay compared to that in the Ultrio Elite assay (χ^2^  = 20.01, *p* < 0.01). HBV NAT yields rate in the MPX 2.0 assay was higher than in the Ultrio and Ultrio Plus assays but lower than that in the Ultrio Elite assay (χ^2^  = 170.10, *p* < 0.01), whereas HCV NAT yields were higher in Ultrio Plus (*p* < 0.01, Fisher’s exact test). Nevertheless, HIV NAT yield rates did not significantly differ between the 5 assays (*p* > 0.05, Fisher’s exact test).

### Ultrio Elite and MPX2.0 assays in ID-NAT mode with similar HBV detection capacity

**Fig. 1:**
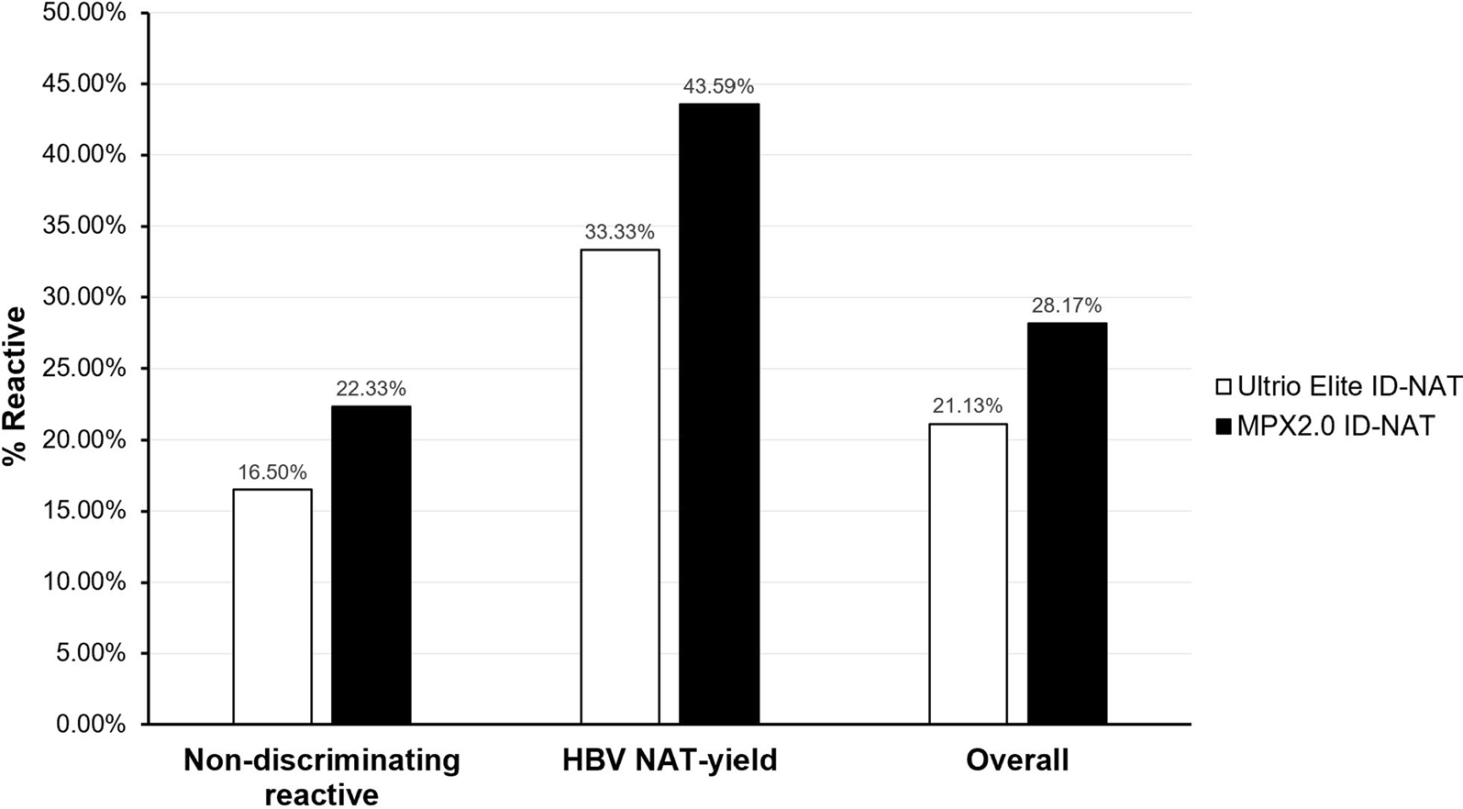
Comparison of HBV-DNA positive results on 103 non-discriminating reactive and 39 HBV NAT-yield samples in the Ultrio Elite ID-NAT (□) and MPX2.0 ID-NAT (■). Non-discriminating reactive indicates 103 screening tests positive but non-discriminating reactive samples, HBV NAT-yield indicates 39 HBV NAT^+^ELISA^−^ yield OBI samples, and overall refers to all 142 specimens

In total, 103 positive screening tests but non-discriminating reactive samples and 39 HBV NAT yield OBI samples were detected using the Ultrio Elite and MPX2.0 assays in ID-NAT mode. All samples were anti-HBc positive with low viral load (< 12 IU/mL and < 20 IU/mL in non-discriminating reactive and HBV NAT yields samples, respectively). Among the 103 non-discriminating reactive samples, the Ultrio Elite and MPX2.0 assays detected 17 (16.50%) and 23 (22.33%) HBV-DNA reactive samples as positive, respectively (Fig. 1). However, the reactive rates did not significantly differ (χ^2^  = 1.12, p > 0.05) between the 2 assays. Out of the 39 HBV NAT-yield samples, Ultrio Elite and MPX2.0 assays detected 13 (33.33%) and 17 (43.59%) HBV-DNA reactive samples, respectively (χ^2^  = 0.87, *p* > 0.05). The overall proportion of HBV-DNA reactive results did not significantly differ between the Ultrio Elite assay (17.54%) and the MPX2.0 system (22.81%). Using the ID-NAT mode, no difference was noted between the Ultrio Elite and MPX2.0 assays in the detection of low HBV loads. However, unlike the Ultrio Elite assay in ID-NAT mode, the MPX2.0 assay in MP-NAT mode might have lower HBV-NAT yields (Table 3).

### Non-discriminating reactive in ID-NAT using the TMA method

Non-discriminating reactive implies reactive donations using NAT screening by the TMA method but not in discriminatory assay. Over the 10 years, 1,763 blood donations (850.99 per million) were non-discriminating reactive (Table 3). The rate of non-discriminating reactive in Shaoxing, Jiaxing, and Huzhou was much lower than that in Xiaoshan, Jiandem, and Yiwu (*p* < 0.05, Additional file 1: Table S2).

**Fig. 2:**
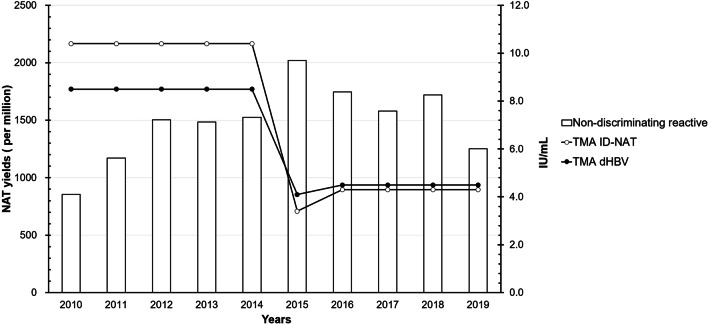
Non-discriminating reactive NAT yields in the blood service centers. The scale at the left indicates NAT yields (per million) in the TMA method, the donations were non-discriminating reactive (□); the scale at the right indicates the LOD (IU/mL) of different reagents in the TMA method in ID-NAT assays (○) and HBV discriminatory assay (●)

Notably, the rate of non-discriminating reactive yields in all donations exhibited a downward trend annually (Additional file 1: Table S2). NAT yields rate of non-discriminating reactive using the TMA method was highest in 2012 and lowest in 2019 (Additional file 1: Table S2). Additionally, the rate of non-discriminating reactive yields in all NAT yields varied across the 3 TMA assays (Fig. 2). In contrast with the Ultrio assay, the Ultrio Plus and Ultrio Elite assays demonstrated better discrimination capacity, which appeared to match the sensitivity of different TMA assays and the gap between screening and discriminatory assays (Fig. 2). The use of Ultrio Elite assay after September 22, 2016, decreased the non-discriminating reactive NAT yields rate because of a smaller screening and discriminatory sensitivity gap than that of the other 2 TMA assays (Fig. 2).

### HBV NAT yields in the blood services

In 10 years, 2202 blood donations were HBV NAT^+^ ELISA^−^. In total, the HBV NAT yield rates exhibited annual fluctuations and varied across blood service centers (Additional file 1: Table S3). HBV NAT yield rate was lowest in Hangzhou (883.58 per million) and highest in Jiande (2581.83 per million). Also, HBV NAT yields rates were associated with TMA assay sensitivity. Therefore, the total HBV NAT yields rates started to increase when the use of the Ultrio Elite assay began in 2017, suggesting that the Ultrio Elite assay in ID-NAT mode had effective HBV screening and discriminating capacities.

### Effects on HBV NAT^+^ELISA^−^ yields in the blood service centers

Analysis of HBV NAT^+^ELISA^−^ yields by demographic groups showed that over 10 years, compared to female donors, overall HBV NAT^+^ELISA^−^ yield prevalence was higher in male donors (Additional file 1: Table S4, χ^2^ = 174.02, *p* < 0.01) and in each blood service except the Xiaoshan (χ^2^ = 2.85, p > 0.05) and Jiande (χ^2^ = 2.51, p > 0.05). Analysis by age group (18–25, 26–35, 36–45, 46–55, > 55) discovered a higher HBV NAT yield rate in the age group 46–55 (χ^2^ = 1796.99, *p* < 0.01) at all blood service centers. Analysis by the level of education revealed that donors with higher education had lower rates of HBV NAT^+^ELISA^−^ yields, which were much higher in the junior high school group (30.88%, χ^2^ = 1042.21, *p* < 0.01), whereas, they were higher in the middle school group in Xiaoshan and Jiande. We found that HBV NAT yields rates were higher in clerk donors (23.30%, χ^2^ = 699.07, *p* < 0.01), except in Jiande and Yiwu, where they were higher in farmer donors. HBV NAT yield prevalence was much higher in first-time donors compared to repeat donors (χ^2^ = 218.70, *p* < 0.05) at all blood service centers. Collectively, these data suggest that risk factors associated with HBV NAT^+^ELISA^−^ yields include male gender, old age (between 46 and 55), low education (middle school and below), lower technology worker including Farmer as well as Worker, and first-time donors.

### HCV and HIV NAT^+^ELISA^−^ yields in blood donors

Among the 2,071,695 blood donations, 2 were HCV NAT^+^ELISA^−^ yield donations whereas 3 were HIV NAT^+^ELISA^−^ yield donations (Table 2). Two HCV NAT^+^ELISA^−^ yield donors were followed up for > 1 year and based on NAT and/or ELISA, none was HCV positive during the follow-up period (Additional file 1: Table S5). All 3 HIV NAT^+^ELISA^−^ yield donors were followed-up and re-sampled after about a month (Additional file 1: Table S6), suggesting that they were in the acute HIV infection phase.

## Discussion

In China, NAT was first used as a pilot project in key blood centers in 2000 [8, 9], including the Blood Center of Zhejiang Province. Herein, we discovered that NAT yield rates for HBV, HCV, and HIV varied over time and between the seven blood service centers. Specifically, the NAT yield rates for HCV (1.54 per million) and HIV (2.31 per million) in Hangzhou were similar to other regions of China (NAT yield range: 0–3.4 per million for HCV [10], 0–3.55 per million for HIV [11]. In our study, the HCV NAT yield rate (0.97 per million) was lower than that in Mediterranean countries with high endemic HCV infection (2.15 per million in Spain, 5.97 per million in Greece, 2.5 per million in Italy, 4.27 per million in Slovenia) [12–15]. HIV NAT yield rate (1.45 per million) was similar to that in the US (0.43 per million) [16] and European countries such as Italy (1.8 per million) and Germany (0.43 per million) [15, 17], but lower than that in HIV-1 endemic countries including South Africa (25.56 per million donations) [18].

In follow-up HCV NAT and serological testing, two HCV NAT-yield cases were negative. Nevertheless, all 3 HIV NAT yield donors were in the acute HIV infection phase. Reports indicate that 15–25% of HCV infections are self-limiting and vary depending on the HCV genotype. According to Lefrère et al., a few immunocompetent HCV-positive patients were found to be negative after self-limiting using ELISA, RIBA, and HCV-RNA test [19]. Therefore, we speculated that these two HCV NAT-yield donors may have had self-limiting HCV infection or were false positives upon HCV NAT tests. Moreover, Akuta et al. [20] reported HBeAg-negative and HBeAb-positive cases where chronic HBV infection persisted while acute HCV infection was spontaneously resolved. In this patient, HCV infection was interestingly accompanied by the appearance of PreC wild type (G1896); an increase in transiently suppressed HBV viral load at a level that was higher than that established before HCV infection. This case was similar to the BD2 case in our study, which was negative in HBV NAT and serological tests and HCV NAT reactive, but HCV-RNA was undetectable one year later and HBV-DNA positive. Therefore, NAT tests employing in HCV low risk population have low positive predictive value, results must be repeated to confirm.

HBV NAT yield rate was much higher than that of HCV and HIV, ranging from 883.58 to 2582.83 per million at different blood service centers (1:387 in Jiande to 1:1132 in Hangzhou). This rate was a little higher than the average figure of China (1:1482, range:1:1861 to 1:1269) [21], and much higher than that in other low HBV endemic countries including USA, Canada, Germany, Switzerland, and New Zealand [22–26], as well as Mediterranean countries with moderate endemism [12–14]. We found that despite common routes of transmission and similar risk factors, the HBV NAT yield rate is higher than that of HCV and HIV, possibly because HBV is highly prevalent in China [5]. Conversely, the extremely low TTI residual risks for HCV and HIV may be attributed to their low prevalence in the population and short window periods of HCV and HIV testing using ID-NAT [16, 27].

Several studies have compared the sensitivity of NAT systems for HBV, HCV, and HIV [28–34]; as a consequence, differing findings have been reported. Using PROCLEIX ULTRIO (Ultrio) assay and TaqScreen multiplex (Cobas MPX) test, Margaritis et al. reported equal HBV NAT yields rate in donations from Hong Kong [29]. However, Phikulsod et al. in Thailand reported that TaqMan MP6 was more sensitive than Ultrio in ID format [30]. Using the Ultrio Plus assay relative to the Ultrio ID-NAT and TaqMan MP6, Marion et al. in South Africa found a significantly higher proportion of replicate assays on HBV NAT yields [28]. In this work, we compared NAT yields rates in five different assays, including Ultrio, Ultrio Plus, and Ultrio Elite assays using the TMA method in ID format, as well as MPX and MPX2.0 using the PCR method in 6MP-format. Consequently, there were no statistically significant differences in HBV, HCV, and HIV NAT yield rates between the 2 NAT methods. Nonetheless, among the 5 assays, Ultrio Plus was effective at non-discriminating reactive and HCV detection, whereas Ultrio Eilte exhibited the highest HBV NAT yield in the screening test. Interestingly, MPX2.0 was slightly but not significantly more sensitive in detecting low viral load OBI samples using the ID format. Collectively, these results indicate that besides reagents sensitivity, the capacity of NAT methods to detect HBV, HCV, and HIV, particularly at low viral loads depends on pool size.

In addition to HBV, HCV, and HIV, some samples were screening tests-positive, but non-discriminating reactive using the TMA method. The reasons for these non-resolved results were potential because of a sensitivity gap between screening and discriminatory reagents in the TMA method, or the viral loads in the donations may have been too low to be detected by discriminatory reagents. Some non-resolved results were found with HBV DNA positive through increasing number of tests, concentrating with high-speed centrifugation, and using other NAT methods [35–37]. In China, Ye et al. [37, 38] found that 91.1% of non-discriminated reactive donors were anti-HBc reactive OBI with low viral loads. Thus, non-discriminating reactive donations have a great risk for HBV transmission and should be excluded. Also, we found that the HBV NAT yields risk factors included male gender, older age, low education level, lower technology work, and first-time donors. Therefore, NAT screening for TTIs and higher sensitivity screening, specifically for HBV, improve the safety of blood supply. Differences in NAT yield at different blood service centers may be attributed to NAT screening methods and virus prevalence in the general population.

## Conclusion

In conclusion, high HBV NAT yield rates were discovered in an analysis of NAT yield rates at seven Chinese blood service centers. Besides, the efficiency of HBV, HCV, and HIV NAT yield was similar for TMA and PCR methods but different in the 5 reagent assays. NAT screening at blood donation reduces the risk of transfusion-transmitted infections, shortens the duration of serological tests, and increases blood safety. Nonetheless, NAT yields rates varied across blood services and hinged on the NAT detection mode and blood donor features.

## Supplementary Information

**Additional file 1: Table S1.** ELISA reagents used for screening donors in HBsAg, anti-HCV, anti-HIV1/2 and anti-TP. **Table S2.** Non-discriminating reactive NAT yields in the seven blood services. **Table S3.** HBV NAT yields rates in the seven blood services. **Table S4.** HBV NAT yields for tested donations with HBsAg negative in the different demographic groups. **Table S5.** The follow-up results of 2 HCV NAT yields blood donors. **Table S6.** The follow-up results of 3 HIV NAT+ELISA- blood donors.

## Data Availability

The data used in this study is available from the corresponding author on reasonable request.

## Abbreviations

ALT: Alanine aminotransferase
CLIA: Chemiluminescent immunoassay
dHBV: Discriminatory test for HBV DNA
dHCV: Discriminatory test for HCV RNA
dHIV: Discriminatory test for HIV RNA
DNA: Deoxyribonucleic acid
ECLIA: Electrochemiluminescence immunoassay
ELISA: Enzyme linked immunosorbent assay
HBcAb: Antibody to hepatitis B core antigen
HBeAb: Antibody to hepatitis B E antigen
HBeAg: Hepatitis B E antigen
HBsAb: Antibody to hepatitis B surface antigen
HBsAg: Hepatitis B virus surface antigen
HBV: Hepatitis B virus
HCV: Hepatitis C virus
HCV-Ab: Antibody to hepatitis C virus
HIV: Human immunodeficiency virus
ID: Individual donation
LOD: Limit of detection
MP: Mini pool
NAT: Nucleic acid amplification testing
OBI: Occult hepatitis B virus infection
PCR: Polymerase chain reaction
RNA: Ribonucleic acid
TMA: Transcription-mediated amplification
TTIs: Transfusion transmission infections
WP: Window period
