# Supplementary material for: The impact of nucleic acid testing to detect human immunodeficiency virus, hepatitis C virus, and hepatitis B virus yields from a single blood center in China with 10-years review
Source: BMC Infect Dis. 2022 Mar 23;22:279. doi: 10.1186/s12879-022-07279-5 (PMC8943971; doi:10.1186/s12879-022-07279-5)
Supplement: Supplementary file 1 — Additional file 1: Table S1. ELISA reagents used for screening donors in HBsAg, anti-HCV, anti-HIV1/2 and anti-TP. Table S2. Non-discriminating reactive NAT yields in the seven blood services. Table S3. HBV NAT yields rates in the seven blood services. Table S4. HBV NAT yields for tested donations with HBsAg negative in the different demographic groups. Table S5. The follow-up results of 2 HCV NAT yields blood donors. Table S6. The follow-up results of 3 HIV NAT+ELISA- blood donors. [file 12879_2022_7279_MOESM1_ESM.docx]

**Additional data**

**Table S1** - ELISA reagents used for screening donors in HBsAg, anti-HCV, anti-HIV1/2 and anti-TP

| **ELISA Reagents Types** | **Company** | **Sensitivity** |
| --- | --- | --- |
| HBsAg | BioMérieux Clinical Diagnostics (Shanghai, China); Ke-Hua BIO-Engineering Co., Ltd. (Shanghai, China); InTec Products, Inc. (Xiamen, China); Abbott Murex (Dartford, UK) | 0.05 IU/mL |
| Anti-HCV | InTec Products, Inc. (Xiamen, China); Ke-Hua BIO-Engineering Co., Ltd. (Shanghai, China); Ortho-Clinical Diagnostics, Inc. (New Jersey, USA) | 0.3 NCU/mL |
| Anti-HIV 1/2 | Livzon Diagnostics Inc. (Zhuhai, China); Beijing Wantai Biological Pharmacy (Beijing, China); BioMérieux Clinical Diagnostics (Shanghai, China); Bio-Rad (Marnes-la-Coquette, France) | 0.4 NCU/mL |
| Anti-TP | Beijing Wantai Biological Pharmacy (Beijing, China); InTec Products, Inc. (Xiamen, China) | 1.5 mIU/mL |

ELISA, Enzyme linked immunosorbent assay; NAT, Nucleic acid amplification testing; HBsAg, Hepatitis B virus surface antigen; HBV, Hepatitis B virus; HCV, Hepatitis C virus; Anti-HCV, Antibody to Hepatitis C virus; HIV, Human immunodeficiency virus; Anti-HIV, Antibody to Human immunodeficiency virus; TP: *Treponema pallidum*; Anti-TP, Antibody to *Treponema pallidum*; IU, International Unit; NCU, National clinical unit.

**Table S2** - Non-discriminating reactive NAT yields in the seven blood services

| **NAT yields**  **(per million)** | | blood services | | | | | | | **Overall** |
| --- | --- | --- | --- | --- | --- | --- | --- | --- | --- |
|  |  | **Hangzhou^&#^** | **Xiaoshan** | **Jiande** | **Yiwu** | **Shaoxing^&#^** | **Jiaxing*^&#^** | **Huzhou*^&#^** |  |
| **Years** | **2010** | 853.73 | / | / | / | / | / | / | 853.73 |
|  | **2011** | 1170.27 | / | / | / | / | / | / | 1170.27 |
|  | **2012** | 1504.11 | / | / | / | / | / | / | 1504.11 |
|  | **2013** | 1156.80 | 4175.37 | 3436.43 | 1726.37 | / | / | / | 1182.63 |
|  | **2014** | 822.50 | 1608.19 | 2532.93 | 1757.78 | / | / | / | 1066.57 |
|  | **2015** | 922.71 | 1845.56 | 2514.93 | 2892.20 | / | / | / | 1241.70 |
|  | **2016** | 515.59 | 1155.57 | 1632.34 | 2745.79 | 313.06 | 311.76 | 553.91 | 641.88 |
|  | **2017** | 542.54 | 1133.95 | 1771.48 | 2029.43 | 1120.47 | 711.87 | 528.05 | 796.69 |
|  | **2018** | 515.17 | 825.45 | 2292.70 | 2790.03 | 692.86 | 474.08 | 330.42 | 707.06 |
|  | **2019** | 378.56 | 426.83 | 1299.73 | 1258.18 | 417.13 | 217.61 | 381.23 | 435.20 |
| **Total** | | 814.19 | 1150.21 | 1998.00 | 2208.33 | 642.57 | 425.42 | 442.11 | 850.99 |

*p<0.05, compared to Xiaoshan; &p<0.01, compared to Jiande; #p<0.01, compared to Yiwu. NAT, Nucleic acid amplification testing.

**Table S3** - HBV NAT yields rates in the seven blood services

| **NAT yields**  **(per million)** | | **blood services** | | | | | | | **TMA proportion** | **In TMA method** | **PCR proportion** | **In PCR method** | **Overall** |
| --- | --- | --- | --- | --- | --- | --- | --- | --- | --- | --- | --- | --- | --- |
|  |  | **Hangzhou** | **Xiaoshan** | **Jiande*** | **Yiwu** | **Shaoxing*** | **Jiaxing** | **Huzhou** |  |  |  |  |  |
| **Years** | **2010** | 811.05 | / | / | / | / | / | / | 100.00% | 811.05 | 0.00% | / | 811.05 |
|  | **2011** | 759.91 | / | / | / | / | / | / | 100.00% | 759.91 | 0.00% | / | 759.91 |
|  | **2012** | 931.11 | / | / | / | / | / | / | 100.00% | 931.11 | 0.00% | / | 931.11 |
|  | **2013** | 655.52 | 0.00 | 0.00 | 431.59 | / | / | / | 79.60% | 624.60 | 20.40% | 738.36 | 647.81 |
|  | **2014** | 710.34 | 1242.69 | 1773.05 | 791.00 | / | / | / | 69.91% | 762.78 | 30.09% | 993.40 | 832.16 |
|  | **2015** | 981.77 | 959.69 | 1886.20 | 876.42 | / | / | / | 61.51% | 901.37 | 38.49% | **1260.41** | 1039.56 |
|  | **2016** | 1009.70 | **1588.91** | 2565.10 | 686.45 | 1324.47 | 599.53 | 627.77 | 36.75% | 1004.75 | 63.25% | **1063.95** | 1042.20 |
|  | **2017** | **1112.90** | 1275.69 | 3381.91 | 1884.47 | 1224.22 | **1305.10** | **1428.84** | 50.40% | **1561.80** | 49.60% | **1081.51** | **1323.58** |
|  | **2018** | 883.15 | 1206.43 | **4126.86** | **2790.03** | 1286.75 | 929.93 | 1081.37 | 41.09% | **1669.50** | 58.91% | 878.48 | **1203.51** |
|  | **2019** | 899.87 | 1219.51 | 1805.18 | 2138.90 | **1611.65** | 1171.76 | 1202.35 | 34.74% | **1529.39** | 65.26% | 1004.61 | **1186.91** |
| **Total** | | 883.58 | 1241.32 | 2581.83 | 1584.23 | 1368.05 | 1024.89 | 1105.28 | 56.01% | 1102.25 | 43.99% | 1012.79 | 1062.90 |

* p<0.05, compared to the Hangzhou. HBV, Hepatitis B virus; NAT, Nucleic acid amplification testing; PCR, Polymerase chain reaction; TMA, Transcription-mediated amplification.

**Table S4** - HBV NAT yields for tested donations with HBsAg negative in the different demographic groups

| **Donor characteristics** | | **Cities/regions** | | | | | | | **Overall** |
| --- | --- | --- | --- | --- | --- | --- | --- | --- | --- |
|  |  | **Hangzhou** | **Xiaoshan** | **Jiande** | **Yiwu** | **Shaoxing** | **Jiaxing** | **Huzhou** |  |
| **Total HBV NAT yields (per million)** | | **883.58** | **1,241.32** | **2,581.83** | **1,584.23** | **1,368.05** | **1,024.89** | **1,105.28** | **1,062.90** |
| **Gender** | **Male** | **706.25 (79.93)** | **888.28 (71.56)** | **1,556.88 (60.30)** | **1,080.16 (68.18)** | **1,051.94 (76.89)** | **783.17 (76.42)** | **821.06 (74.29)** | **807.55 (75.98)** |
|  | **Female** | 177.33 (20.07) | 353.03 (28.44) | 1024.95 (39.70) | 504.07 (31.82) | 316.1 (23.11) | 241.72 (23.58) | 284.21 (25.71) | 255.35 (24.02) |
| **Age (years)** | **18-25** | 85.58 (9.69) | 102.49 (8.26) | 25.95 (1.01) | 24.00 (1.52) | 25.91 (1.89) | 87.02 (8.49) | 102.63 (9.29) | 77.23 (7.27) |
|  | **26-35** | 163.45 (18.50) | 182.21 (14.28) | 129.74 (5.03) | 156.02 (9.85) | 243.55 (17.80) | 217.55 (21.23) | 228.95 (20.71) | 179.56 (16.89) |
|  | **36-45** | 309.18 (34.99) | 375.81 (30.28) | 817.36 (31.66) | 456.07 (28.79) | 445.65 (32.58) | 314.23 (30.66) | 347.37 (31.43) | 352.37 (33.15) |
|  | **46-55** | **312.26 (35.34)** | **558.02 (44.95)** | **1,414.17 (54.77)** | **912.13 (57.58)** | **606.29 (44.32)** | **401.25 (39.15)** | **394.74 (35.71)** | **429.12 (40.37)** |
|  | **>55** | 13.11 (1.48) | 22.78 (1.83) | 194.61 (7.54) | 36.01 (2.27) | 46.64 (3.41) | 4.83 (0.47) | 31.58 (2.86) | 24.62 (2.32) |
| **Education** | **Primary school** | 26.99 (3.05) | 56.94 (4.59) | 103.79 (4.02) | 144.02 (9.09) | 62.18 (4.55) | 82.18 (8.02) | 102.63 (9.29) | 49.24 (4.63) |
|  | **Junior high school** | **254.43 (28.80)** | 125.27 (10.09) | 609.78 (23.62) | **696.10 (43.94)** | **528.56 (38.64)** | **391.58 (38.21)** | **402.64 (36.43)** | **328.23 (30.88)** |
|  | **Middle school** | 154.97 (17.54) | **375.81 (30.28)** | **1,180.64 (45.73)** | 348.05 (21.97) | 316.10 (23.11) | 222.38 (21.70) | 300.00 (27.14) | 240.87 (22.66) |
|  | **College** | 114.88 (13.00) | 239.15 (19.27) | 311.38 (12.06) | 288.04 (18.18) | 186.55 (13.64) | 212.71 (20.75) | 213.16 (19.29) | 156.88 (14.76) |
|  | **Undergraduate** | 98.69 (11.17) | 113.88 (9.17) | 103.79 (4.02) | 60.01 (3.79) | 155.46 (11.36) | 58.01 (5.66) | 55.26 (5.00) | 96.54 (9.08) |
|  | **Graduate and above** | 8.48 (0.96) | 11.39 (0.92) | 12.97 (0.50) | 12.00 (0.76) | 15.55 (1.14) | 4.83 (0.47) | 7.89 (0.71) | 9.17 (0.86) |
|  | **Missing** | 225.14 (25.48) | 318.87 (25.69) | 259.48 (10.05) | 36.01 (2.27) | 103.64 (7.58) | 53.18 (5.19) | 23.68 (2.14) | 181.98 (17.12) |
| **Occupation** | **Farmer** | **64.77 (7.33)** | **204.99 (16.51)** | **1,193.61 (46.23)** | **324.05 (20.54)** | **243.55 (17.80)** | 67.68 (6.60) | **228.95 (20.71)** | **150.12 (14.12)** |
|  | **Worker** | 58.60 (6.63) | 148.05 (11.93) | 181.64 (7.04) | 84.01 (5.30) | 191.73 (14.02) | **256.22 (25.00)** | 126.32 (11.43) | 104.26 (9.81) |
|  | **Clerk** | **195.84 (22.16)** | **398.59 (32.11)** | **207.58 (8.04)** | **216.03 (13.64)** | **393.83 (28.79)** | **348.07 (33.96)** | **331.58 (30.00)** | **247.62 (23.30)** |
|  | **Student** | 28.53 (3.23) | 34.16 (2.75) | 38.92 (1.51) | 36.01 (2.27) | 67.37 (4.92) | 43.51 (4.25) | 31.58 (2.86) | 34.75 (3.27) |
|  | **Military** | 6.94 (0.79) | 0.00 (0.00) | 12.97 (0.50) | 0.00 (0.00) | 5.18 (0.38) | 4.83 (0.47) | 15.79 (1.43) | 6.76 (0.64) |
|  | **Government employee** | 25.44 (2.88) | 56.94 (4.59) | 220.56 (8.54) | 60.01 (3.79) | 67.37 (4.92) | 38.67 (3.77) | 47.37 (4.29) | 41.99 (3.95) |
|  | **Medical staff** | 8.48 (0.96) | 11.39 (0.92) | 77.84 (3.02) | 48.01 (3.03) | 31.09 (2.27) | 19.34 (1.89) | 31.58 (2.86) | 17.38 (1.63) |
|  | **Others** | 494.99 (56.02) | 387.20 (31.19) | 648.70 (25.13) | 816.12 (51.52) | 367.92 (26.89) | 246.55 (24.06) | 292.11 (26.43) | 460.01 (43.28) |
| **Donor status** | **First time** | **524.29 (59.34)** | **876.89 (70.64)** | **1,621.75 (62.81)** | **1,164.17 (73.48)** | **860.21 (62.88)** | **681.65 (66.51)** | **686.85 (62.14)** | **662.74 (62.35)** |
|  | **Repeat** | 359.29 (40.66) | 364.42 (29.36) | 960.08 (37.19) | 420.06 (20.52) | 507.84 (37.12) | 343.24 (33.49) | 418.43 (37.66) | 400.16 (37.65) |

Data are shown as number (%), indicate the percentages of different demographic groups in NAT^+^ELISA^-^ yields. HBV, Hepatitis B virus; HBsAg, Hepatitis B virus surface antigen; ELISA, Enzyme linked immunosorbent assay; NAT, Nucleic acid amplification testing.

**Table S5** - The follow-up results of 2 HCV NAT yields blood donors

| **Donors** | **Sampling type** | **Sampling date** | **Donation type** | **Age** | **Gender** | **Times of Donation** | **TMA** | | **PCR** | **ELISA** | | **CLIA** | | | | | | |
| --- | --- | --- | --- | --- | --- | --- | --- | --- | --- | --- | --- | --- | --- | --- | --- | --- | --- | --- |
|  |  |  |  |  |  |  | **First NAT result** | **Discriminatory test result** | **ID-NAT result** | **HCV-Ab** | **HBsAg** | **HCV-Ab** | **HBsAg** | **HBsAb** | **HBeAb** | **HBeAg** | **HBcAb** | **HBcAb-IgM** |
| **BD1** | Donation | September 16, 2015 | WB | 34 | Male | 3WB | **Pos** | **Pos(HCV)** | / | Neg | Neg | / | / | / | / | / | / | / |
|  | Follow-up | September 11, 2016 |  |  |  |  | Neg | Neg | Neg | Neg | Neg | Neg | Neg | **Pos** | Neg | Neg | Neg | Neg |
| **BD2** | Donation | May 26, 2016 | PLT | 33 | Male | 10WB and 5PLT | **Pos** | **Pos(HCV)** | / | Neg | Neg | / | / | / | / | / | / | / |
|  | Follow-up | January 11, 2018 |  |  |  |  | Neg | Neg | Pos(HBV) | Neg | Neg | Neg | Neg | **Pos** | **Pos** | Neg | Neg | Neg |

BD, blood donor; WB, whole blood; PLT, platelet; HBV, Hepatitis B virus; HCV, Hepatitis C virus; HBcAb, Antibody to hepatitis B core antigen; HBeAb, Antibody to hepatitis B E antigen; HBeAg, Hepatitis B E antigen; HBsAb, Antibody to hepatitis B surface antigen; HBsAg, Hepatitis B virus surface antigen; HCV-Ab, Antibody to hepatitis C virus; ID, individual donation; /, not applicable; NAT, nucleic acid amplification testing; Neg, negative; Pos, positive; CLIA, chemiluminescence immunoassay; ELISA, enzyme linked immunosorbent assay. TMA, transcription-mediated amplification; PCR, polymerase chain reaction.

**Table S6** - The follow-up results of 3 HIV NAT^+^ELISA^-^ blood donors

| **Donors** | **Donation date** | | **Donation type** | **Age** | **Gender** | **Times of Donation** | **NAT** | | | | | | **ELISA** | | **Confirmatory assay** |
| --- | --- | --- | --- | --- | --- | --- | --- | --- | --- | --- | --- | --- | --- | --- | --- |
|  |  |  |  |  |  |  | **First NAT result (TMA, S/Co)** | **Discriminatory test result (TMA, S/Co)** | **Method for the NAT test** | **6MP-NAT result for ROCHE screening test (Ct)** | **ID-NAT result for ROCHE confirmatory test (Ct)** | **Method for the NAT test** | **antibody to HIV (reagent 1)** | **antibody to HIV and p24 antigen (reagent 2)** | **Western blot** |
| **BD3** | X | May 25, 2012 | WB | 25 | Male | 1WB | **Pos, 18.46** | **Pos, 32.61** | ULTRIO | / | / | / | neg | neg | neg |
|  | X+32* | June 26, 2012 |  |  |  |  | / | **Pos, 31.85** | ULTRIO | / | / | / | **pos** | **pos** | **gp160,gp120,gp41,p24** |
| **BD4** | X | September 17, 2015 | WB | 25 | Male | 1WB | **Pos, 14.15** | / | PLUS | **Pos, 27.0** | **Pos, 24.4** | MPX2.0 | neg | neg | neg |
|  | X+23 | October 10, 2015 |  |  |  |  | / | **Pos, 27.06** | PLUS | / | / | / | **pos** | **pos** | **gp160,gp120,gp41,p24** |
| **BD5** | X | July 8, 2016 | WB | 50 | Male | 2WB | **Pos, 13.83** | **Pos, 26.65** | ELITE | / | / | / | neg | neg | neg |
|  | X+35 | August 12, 2016 |  |  |  |  | / | / | ELITE | / | **Pos, 21.9** | MPX2.0 | **pos** | **pos** | **gp160,gp120,gp41,p24** |

*Days from index donation.

BD, blood donor; WB, whole blood; HIV, Human immunodeficiency virus; ID, individual donation; MP, mini-pool; /, not applicable; NAT, nucleic acid amplification testing; ELISA, enzyme linked immunosorbent assay; Neg, negative; Pos, positive; ULTRIO, Procleix^®^ Ultrio^®^ assay; PLUS, Procleix^®^ Ultrio Plus^®^ assay; ELITE, Procleix^®^ Ultrio Elite^®^ Assay; MPX, Cobas^®^ TaqScreen MPX Test; MPX2.0, Cobas^®^ TaqScreen MPX Test, version 2.0.
